# Rhizosphere Bacterial Community Structure and Predicted Functional Analysis in the Water-Level Fluctuation Zone of the Danjiangkou Reservoir in China During the Dry Period

**DOI:** 10.3390/ijerph17041266

**Published:** 2020-02-16

**Authors:** Zhao-Jin Chen, Yang Shao, Ying-Jun Li, Li-An Lin, Yan Chen, Wei Tian, Bai-Lian Li, Yu-Ying Li

**Affiliations:** 1 Innovation Center of Water Security for Water Source Region of Mid-route Project of South-North Water Diversion of Henan Province, School of Agricultural Engineering, Nanyang Normal University, Nanyang 473061, China; zhaojin_chen@163.com (Z.-J.C.); 17613760587@163.com (Y.S.); li191388348@163.com (Y.-J.L.); linlian198208@163.com (L.-A.L.); yanna_chen@yeah.net (Y.C.); 2 Nanjing Institute of Environmental Sciences, Ministry of Ecology and Environment, Nanjing 210042, China; 3 Ecological Complexity and Modelling Laboratory, Department of Botany and Plant Sciences, University of California, Riverside, CA 92521, USA; bai-lian.li@ucr.edu

**Keywords:** water-level fluctuation zone, Danjiangkou reservoir, revegetation, bacterial community structure and function, PICRUSt analysis

## Abstract

The water-level fluctuation zone (WLFZ) is a transitional zone between terrestrial and aquatic ecosystems. Plant communities that are constructed artificially in the WLFZ can absorb and retain nutrients such as nitrogen (N) and phosphorus (P). However, the microbial community composition and function associated with this process have not been elucidated. In this study, four artificially constructed plant communities, including those of herbs (*Cynodon dactylon* and *Chrysopogon zizanioides*), trees (*Metasequoia glyptostroboides*), and shrubs (*Salix matsudana*) from the newly formed WLFZ of the Danjiangkou Reservoir were evaluated. The bacterial community compositions were analyzed by 16S rRNA gene sequencing using a MiSeq platform, and the functions of these communities were assessed via Phylogenetic Investigation of Communities by Reconstruction of Unobserved States (PICRUSt) analysis. The results showed that the bacterial communities primarily comprised 362 genera from 24 phyla, such as Proteobacteria, Acidobacteria, Actinobacteria, and Gemmatimonadetes, showing the richness of the community composition. Planting altered the bacterial community composition, with varying effects observed among the different plant types. The bacterial community functional analysis revealed that these bacteria were primarily associated with six biological metabolic pathway categories (e.g., metabolism, genetic information processing, and environmental information processing) with 34 subfunctions, showing the richness of community functions. The planting of *M. glyptostroboides*, *S. matsudana*, and *C. dactylon* improved the metabolic capabilities of bacterial communities. N- and P-cycling gene analysis showed that planting altered the N- and P-cycling metabolic capacities of soil bacteria. The overall N- and P-metabolic capacity was highly similar between *C. dactylon* and *C. zizanioides* samples and between *S. matsudana* and *M. glyptostroboides* samples. The results of this study provide a preliminary analysis of soil bacterial community structure and function in the WLFZ of the Danjiangkou Reservoir and provides a reference for vegetation construction in this zone.

## 1. Introduction

The water-level fluctuation zone (WLFZ) is an alternating submerged and exposed zone formed in the areas surrounding rivers, reservoirs, and lakes due to seasonal or periodic water-level fluctuations. The WLFZ plays an important role in connecting water and land, while also acting as the final barrier for water quality safety in reservoirs [1,2]. Since the Middle Route of the South-to-North Water Diversion Project (MR-SNWDP) was launched in December 2014, the normal storage level of the Danjiangkou Reservoir had been elevated from 157 to 170 m. The newly inundated farmland area comprises nearly 1.73 × 10^8^ m^2^ and a new WLFZ formed [3,4]. Long-term monitoring indicates that due to pollution from agricultural nonpoint sources and rural domestic sewage, the Danjiangkou Reservoir receives large nitrogen (N) and phosphorus (P) loads. The potential risk of releasing nutrients from the newly inundated area of the reservoir cannot be ignored [5,6,7,8].

Studies have shown that the presence of plants and soil microbes in the WLFZ increase the absorption and retention of nutrients, such as soil N and P, and promoting their presence is an effective approach for the control of N and P levels and prevention of eutrophication of the Danjiangkou Reservoir [9,10,11]. Microbes are the primary drivers in the biogeochemical cycling of soil elements [12]. Currently, there is a dearth of studies on microbial community composition and function during plant absorption and retention of nutrient elements, such as N and P, in the WLFZ. Ye et al. [13] investigated the bacterial and fungal community compositions in natural recovery and artificial revegetation areas in the WLFZ of China’s Three Gorges Reservoir Area, observing no significant differences between the two areas using traditional plate cultivation and isolation methods. Additionally, Wang et al. [10] analyzed the abundance and community composition of bacteria capable of nitrite-dependent anaerobic methane oxidation in the WLFZ of the Three Gorges Reservoir Area using a clone library and quantitative polymerase chain reaction (qPCR) methods. Compared with the traditional plate cultivation/isolation and clone library techniques, high-throughput sequencing enables the high-throughput acquisition of specific DNA fragments to allow for a more complete evaluation of the composition and structure of microbial communities [14]. This method has been used worldwide to investigate the composition and distribution patterns of various soil microbial communities as well as their influencing factors [15,16,17,18]. However, this method has not been extensively used to study the rhizosphere microbial community composition in WLFZs [19]. Moreover, available high-throughput sequencing data have been primarily used to perform microbial community structure analyses (alpha and beta diversity) rather than functional studies. Phylogenetic Investigation of Communities by Reconstruction of Unobserved States (PICRUSt) is a bioinformatics software package for predicting microbial community function and metabolism. Additionally, PICRUSt can predict the metabolic function profile of the corresponding bacteria based on 16S rRNA gene sequences. This method is convenient, fast, and cheap, and its prediction results have high reliability [20,21,22,23]. PICRUSt has been used to study the function of soil bacteria associated with different plants, such as barley, tomato [24], *Suaeda salsa* [25], *Sedum alfredii* [26], and *Elsholtzia splendens* [27], laying the foundation for further investigations of the ecological functions of bacteria in these habitats.

Currently, few studies have reported the combined use of high-throughput sequencing and PICRUSt functional prediction analysis to study soil microbes in WLFZs. The use of these two methods could help to elucidate the microbial community composition and function in this environment. In the present study, we analyzed the soil bacterial community structure in different artificially constructed plant communities of herbs (*Cynodon dactylon* and *Chrysopogon zizanioides*), trees (*Metasequoia glyptostroboides*), and shrubs (*Salix matsudana*) by 16S rRNA gene sequencing using an Illumina MiSeq platform, examining differences between the bacterial community structure associated with these plants and that of an unplanted (bare) control in the newly formed WLFZ of the Danjiangkou Reservoir. Additionally, we predicted the function of rhizosphere bacteria by PICRUSt analysis and examined differences in the bacterial community function. The results of this study preliminarily elucidated the soil bacterial community structure and function and provide a reference for vegetation construction and water environment protection for this site.

## 2. Materials and Methods 

### 2.1. Study Site, Sampling, and Soil Physicochemical Properties

With the Dangjiangkou Dam fully functioning in 2014, the water level in the reservoir fluctuates from 145 m in summer to 170 m in winter. This study was conducted in the Caijiadu, located in the Xijiadian town of Dangjiangkou city, Hubei province, China (32°34’39.45” N, 111°30’45.15” E). After the original surface soil (10 cm thick topsoil) was removed, vegetation restoration was carried out along the elevation from 155 m to 170 m in March 2013 [6]. Herbs (*C. dactylon* and *C. zizanioides*), trees (*M. glyptostroboides*), and shrubs (*S. matsudana*) were planted between the elevations of 155 and 170 m. Samplings were conducted on 10 June 2017 when the water level of the study area was about 145 m. Rhizosphere soil (three biological replicates for each sample) was obtained by firstly gently shaking off the loosely bound soil, while the rhizosphere soil adhering to the root system was isolated by more vigorous shaking or by hand [28]. Unplanted (bare) soil were collected from nonplant sites. Each sample was divided into two parts. One part was immediately sieved through a 2 mm mesh and then stored at 4 °C until soil nitrate–nitrogen (NO_3_^−^–N) could be analyzed, the other section was air-dried for analysis of physicochemical soil properties. Soil pH was determined with a pH meter (PHS-3C, Leizi, China) in 1:2.5 (soil: water, weight/volume, air-dried soil) suspensions. Soil organic carbon (SOC) and total nitrogen (TN) were analyzed with an N/C Soil Analyzer (Flash, EA, 1112 Series, Italy). Exchangeable ammonium nitrogen (NH_4_^+^–N) and NO_3_^−^–N were determined with a spectrophotometer using the indophenol blue colorimetric method and phenol disulfonic acid colorimetry, respectively. Soil total phosphorus (TP) and total potassium (TK) were determined by the molybdenum (Mo)–antimony (Sb) colorimetric method and flame atomic absorption spectrophotometry.

### 2.2. DNA Extraction and Sequencing

Genomic DNA was extracted from 0.5 g of fresh soil using the Fast DNA^®^ SPIN for Soil Kit (MP Biochemicals, Solon, OH, USA) according to the manufacturer’s instructions. Electrophoresis and Nano Drop ND 2000 (Thermo Scientific, USA) were used to examine the quantity of extracted DNA. The V3–V4 region of the bacterial 16S rRNA gene was amplified using 338F (5′-ACTCCTACGGGAGGCAG CA-3′) and 806R (5′-GGACTACHVGGGTWTCTAAT-3′) with sample-identifying barcodes. The PCR assays were performed in a 20 μL mixture containing 4 μL of 5 × FastPfu buffer, 2 μL of 2.5 mM dNTPs, 0.8 μL of each primer (5 μM), 0.4 μL of FastPfu Polymerase, 10 ng of template DNA, and Milli-Q water. The PCR conditions were as follows: 95 °C for 3 min; followed by 27 cycles at 95 °C for 30 s, 55 °C for 30 s, and 72 °C for 45 s; and a final extension at 72 °C for 10 min. PCR was performed in triplicate for each sample, and the products were purified using the AxyPrepDNA Gel Extraction Kit (Axygen Biosciences, Union city, CA, USA) and requantified with QuantiFluor™ ST (Promega, Madison, WI, USA). The sequencing was performed by Shanghai Majorbio Bio-Pharm Technology Co., Ltd. (Shanghai, China) with an Illumina MiSeq PE300 platform. 

### 2.3. Bioinformatics Analysis

After sequencing, the raw data were filtered according to barcode and primer sequences using the software of Trimmomatic and FLASH as follows: bases lower than 20 on the reads tail were filtered; the minimum overlap was 10 bp when merging the paired reads; the maximum mismatch ratio was 0.2 in the overlap and the maximum barcode and primer mismatch number were set as 0 and 2, respectively. Then the high-quality sequences were processed using the QIIME software package (V1.7.0) [29]. Sequence analysis was performed in UPARSE (V7.0.1001) [30]. Sequences with ≥97% similarity were assigned to same operational taxonomic units (OTUs). For each representative sequence, the Greengenes Database was used to annotate taxonomic information [31]. Community richness and diversity were analyzed using alpha diversity estimators, including the abundance-based coverage estimator (ACE), Chao1, Simpson, and Shannon indexes, calculated on Mothur (version 1.30, https://www.mothur.org/) and through principal coordinates analysis (PCoA) and the unweighted pair group method with arithmetic mean (UPGMA) cluster analysis of beta diversity using QIIME software. Functions of the 16S rRNA were predicted based on the information in the Kyoto Encyclopedia of Genes and Genomes (KEGG) (http://www.genome.jp/kegg/) database [32]. The OTU abundance table was standardized, and then the KEGG ortholog cluster information was obtained and calculated from the Greengenes ID of each OTU, using the PICRUSt software version 1.1.1 (http://picrust.github.io/picrust/) [20]. Heatmaps were generated from the gene copy number of the functional genes and log10 transformed using the program Heml heatmap illustrator [33]. The bacterial sequencing data were uploaded into the Sequence Read Archive (SRA) of NCBI (http://www.ncbi.nlm.nih.gov/sra) and can be accessed through accession number PRJNA604313.

### 2.4. Statistical Analyses

The data of the treatments were compared by analysis of variance and Tukey’s test at 5% significance level (*p* < 0.05) in SPSS V. 19.0 (IBM Corp., Armonk, NY, USA) for Windows. 

## 3. Results

### 3.1. Soil Physicochemical Properties in Rhizosphere of Plants in the WLFZ of the Danjiangkou Reservoir

The physicochemical properties were measured for planted and bare soil samples collected from the four artificially planted communities in the WLFZ of the Danjiangkou Reservoir. Table 1 shows that the soil pH values were similar among the five groups of samples, ranging from 7.15 to 8.61. The TN in the bare soil samples was higher than those of the planted samples. The TN concentrations in the bare soil and *M. glyptostroboides* soil samples were greater than 1.00 g/kg. The NO_3_^−^–N in the bare soil samples were 123.80 mg/kg, higher than *C. zizanioides* and *M. glyptostroboides* soil samples. The NH_4_^+^–N in the bare soil samples were 79.35 mg/kg, higher than *C. dactylon*, *C. zizanioides*, and *S. matsudana* soil samples. The TP concentrations observed for the bare soil samples were 0.79 g/kg, significantly higher than those of *C. zizanioides*, *M. glyptostroboides*, and *S. matsudana* soil samples (*p* < 0.05). The TK concentrations observed for the bare soil samples were 4.95 g/kg, significantly higher than those of *C. dactylon*, *C. zizanioides*, and *S. matsudana* soil samples (*p* < 0.05). Similarly, the soil SOC of the planted samples was significantly higher than that of the bare soil samples (*p* < 0.05).

### 3.2. Rhizosphere Bacterial Community Structure Analysis in Different Samples

#### 3.2.1. High-Throughput Sequencing Data and Diversity Assessment

**Figure 1:**
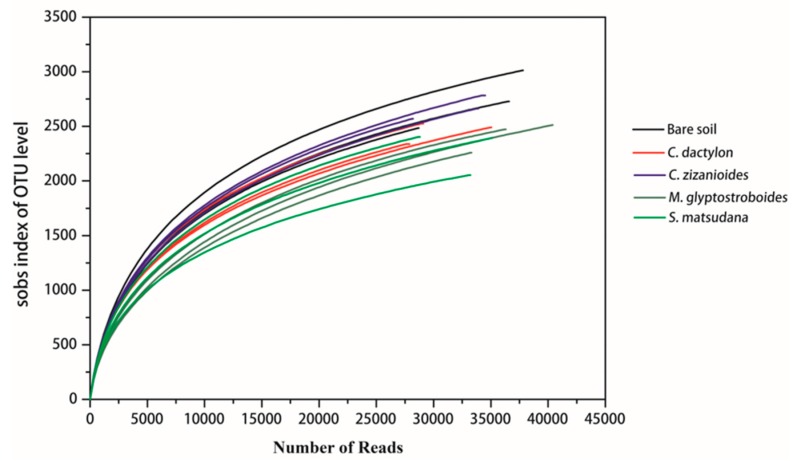
Rarefaction curves based on the sequencing results.

Rarefaction curves used to assess the sampling depth of the samples and to evaluate whether the number of sequences was sufficient to cover all groups. For each of the soil samples in the WLFZ of the Danjiangkou Reservoir, 28,144–40,393 sequences were obtained and the rarefaction curve is shown in Figure 1. As the sequencing data increased, the species richness increased during the early stage, while the species number generally stabilized when the sequence number reached 20,000 or higher. This result indicates that the sequencing data were reasonable and could represent species richness. The community richness indices Chao1 and ACE, as well as the community diversity indices Shannon, Simpson, and library coverage were used for community evaluations. The samples showed rich community composition with high community diversity (Table 2). Considering the total number of species observed (sobs), the richness indices (Chao1 and ACE), the diversity indices (Simpson and Shannon) and library coverage, the soil bacterial community diversity decreased in the following order: *C. zizanioides* > Bare soil > *C. dactylon* > *M. glyptostroboides* > *S. matsudana*.

#### 3.2.2. Bacterial Community Composition and Beta Diversity Analysis

**Figure 2:**
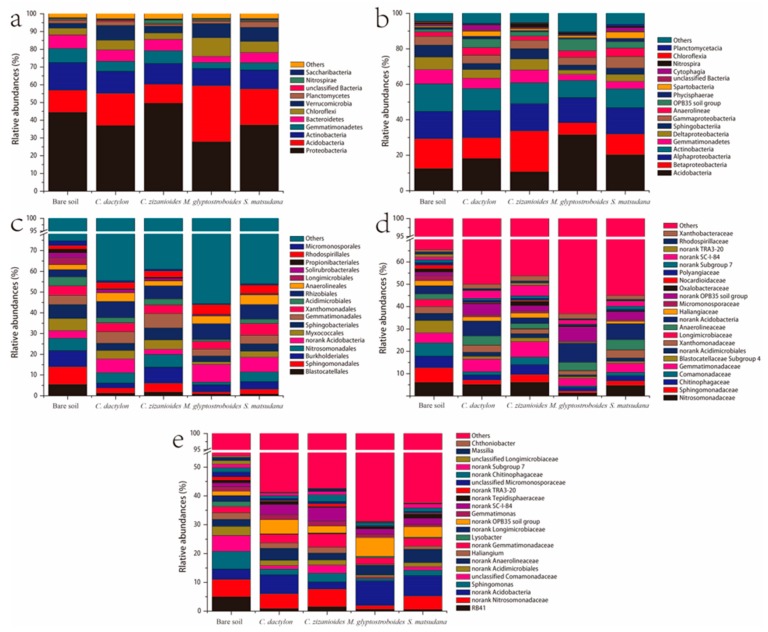
Relative abundance of bacterial sequences at the phylum (**a**), class (**b**), order (**c**), family (**d**) and genus (**e**) levels.

The high-throughput sequencing results showed that the soil bacteria consisted primarily of 24 phyla in the WLFZ of the Danjiangkou Reservoir, including Proteobacteria, Acidobacteria, Actinobacteria, Gemmatimonadetes, Bacteroidetes, Chloroflexi, Verrucomicrobia, and Planctomycetes (Figure 2). The sum of the OTUs affiliated with Proteobacteria, Acidobacteria, Actinobacteria, Gemmatimonadetes, Bacteroidetes, and Chloroflexi accounted for 85.12%–91.96% of the total sequences, indicating that members of these phyla were dominant in the bacterial communities. Proteobacteria was the most abundant phylum in the *C. dactylon*, *C. zizanioides*, *S. matsudana*, and bare soil samples, accounting for 36.98%–49.58% of the total sequences. In addition, Proteobacteria was the second most abundant phylum in the *M. glyptostroboides* soil samples, accounting for 27.87% of the total OTUs. Acidobacteria was the second most abundant phylum in the *C. dactylon* and *S. matsudana* soil samples, accounting for 18.13% and 20.28% of the total OTUs, respectively. Actinobacteria was the second most abundant phylum in the *C. zizanioides* and bare soil samples, accounting for 15.54% and 11.81% of the total OTUs, respectively. Further analysis of the bacteria from other taxa showed that bare soil samples comprised 17 phyla, 30 classes, 67 orders, 137 families, and 219 genera. *C. dactylon* soil samples comprised 19 phyla, 35 classes, 72 orders, 136 families, and 195 genera. *C. zizanioides* soil samples comprised 20 phyla, 36 classes, 75 orders, 141 families, and 206 genera. *M. glyptostroboides* soil samples comprised 21 phyla, 36 classes, 75 orders, 120 families, and 163 genera. *S. matsudana* samples comprised 17 phyla, 33 classes, 71 orders, 129 families and 179 genera. 

**Figure 3:**
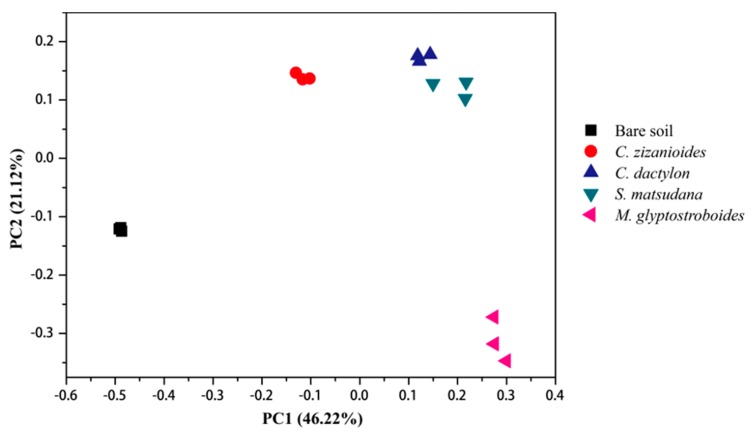
Principal coordinates analysis (PCoA) results of bacterial community diversity.

**Figure 4:**
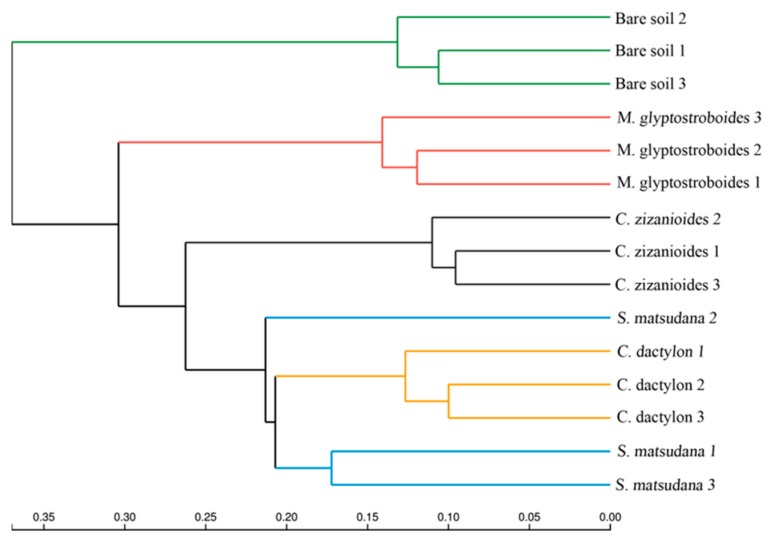
Unweighted pair group method with arithmetic mean (UPGMA) clustering tree based on the Bray–Curtis distance matrix. The digital number represented three biological replicates for each sample.

Both principal coordinates analysis (PCoA) and unweighted pair group method with arithmetic mean (UPGMA) cluster analysis can describe the community differences between various samples, where a short distance between two samples indicates a similar species composition in the two samples. The PCoA results (at OTU level) of rhizosphere bacterial communities associated with plants from the WLFZ of the Danjiangkou Reservoir are shown in Figure 3. In the PCoA plot, the *M. glyptostroboides* samples clustered in the bottom-right, *C. zizanioides* samples were distributed in the upper-middle, the *C. dactylon* and *S. matsudana* samples were close to each other and distributed in the upper-right area, and the bare soil samples were the farthest from the four groups of planted soils and were distributed in the left. The results of the UPGMA cluster analysis based on the Bray–Curtis distance matrix were similar to the PCoA results. The five groups of soil samples were divided into four classes at a similarity level of 0.22. The *C. dactylon* and *S. matsudana* soil samples clustered together, while the bare soil samples were the farthest from the other samples (Figure 4). The above results indicate that artificial vegetation construction affected soil bacterial communities in the WLFZ of the Danjiangkou Reservoir, with varying effects observed among the different plant species.

#### 3.2.3. Correlation Analysis of Bacterial Community and Environmental Factors

**Figure 5:**
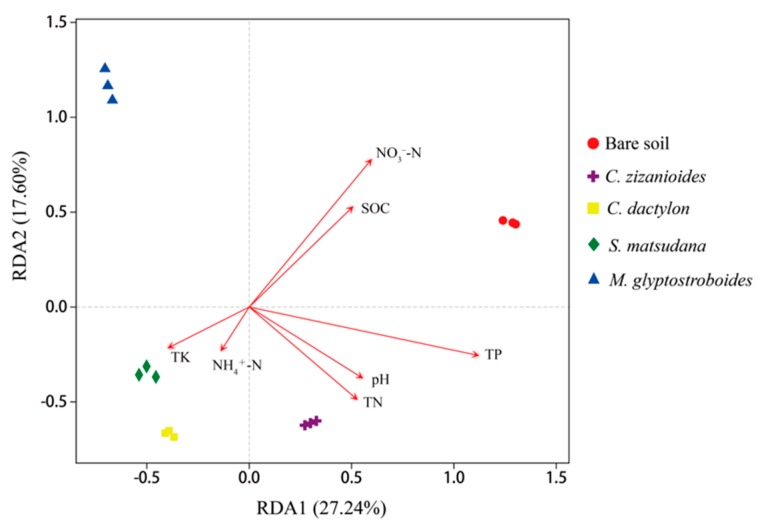
Redundancy analysis (RDA) of bacterial communities and physicochemical characteristics of soil.

Canoco 4.5 (Biometris, Wageningen, The Netherlands) was used for redundancy analysis (RDA) of the bacterial communities at the OTU level. The RDA analysis results are shown in Figure 5. The physicochemical properties with a high correlation with the first ordination axis were TP (R = 0.8398, *p* = 0.001), while NO_3_^−^–N exhibited a high correlation with the second ordination axis (R = 0.8005, *p* = 0.009). These environmental factors had extremely significant correlation with the bacterial community composition (*p* < 0.01). 

#### 3.2.4. Analysis of Different Bacteria From Different Samples

**Figure 6:**
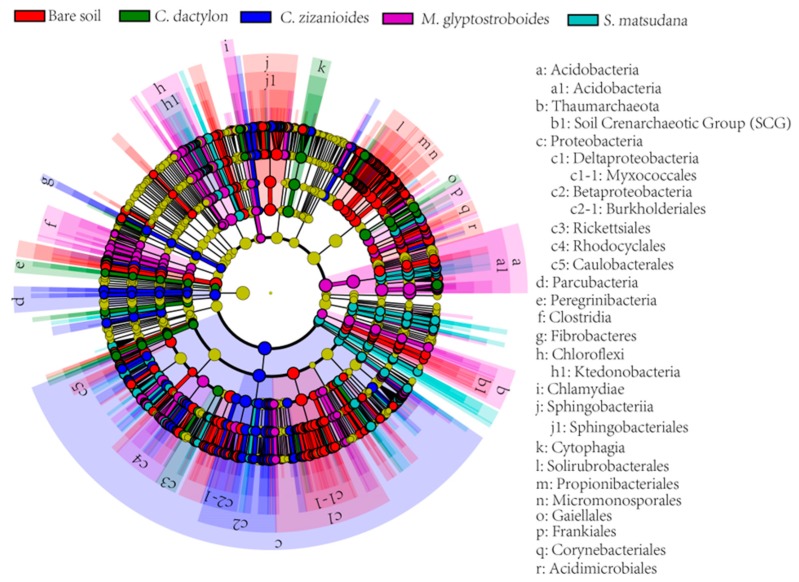
Identification of the most differentially abundant taxa between different samples by using the online statistical linear discriminant analysis effect size (LEfSe) tool.

To determine the differences in bacterial species richness among the different groups, we used the online statistical linear discriminant analysis effect size (LEfSe) tool to search for metagenomic biomarkers. Differential bacterial taxa in different samples were calculated using the LEfSe method; information on the differences among all bacteria at the phylum, class, order, family, and genus levels are illustrated in a pie chart (Figure 6). At the phylum level, significant differences were found in Acidobacteria, Chlamydiae, Ignavibacteriae, Thaumarchaeota in the *M. glyptostroboides* soil samples; Gemmatimonadetes, Peregrinibacteria in the bare soil samples; Microgenomates in the *C. dactylon* soil samples; Fibrobacteres, Parcubacteria, Proteobacteria in the *C. zizanioides* soil samples; and TM6__Dependentiae in the *S. matsudana* soil samples.

**Figure 7:**
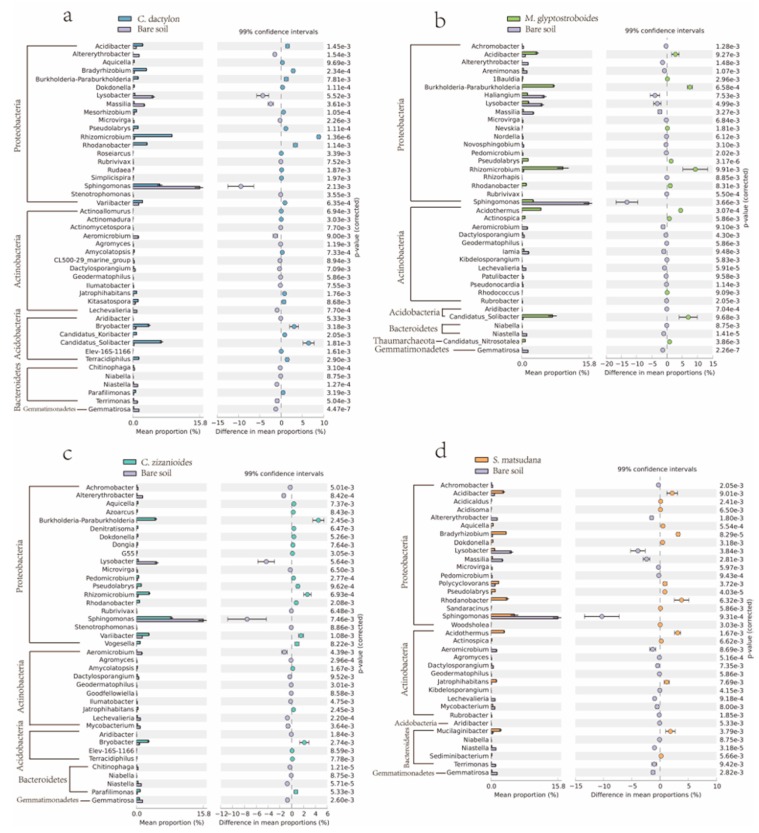
Extended error bar plots showing statistically significant differences in the bacterial community composition at the genus levels between different planted soils (**a**: *C. dactylon*, **b**: *M. glyptostroboides*, **c**: *C. zizanioides* and **d**: *S. matsudana*) and the bare control. Error bars indicate within-group standard deviations. Presented categories passed a corrected *p* value of <0.01 in Welch’s t test.

To study the effects of artificial vegetation construction on soil bacterial communities, we used the software STAMP [34] to comparatively analyze differences in the bacterial community composition at the genus levels between different planted soils and the bare control. Observed differences in the community compositions at the genus level are shown in Figure 7. A total of 127 bacterial genera indicated extremely significant differences (*p* < 0.01) among the four groups of planted soil samples, when compared with the bare soil control. These differentially observed bacteria were distributed among nine phyla, most of which were affiliated with Proteobacteria, Actinobacteria, Acidobacteria, and Bacteroidetes. Among these differentially observed bacterial genera, three or more planted treatments significantly increased the proportions of *Burkholderia*, *Paraburkholderia*, *Dokdonella*, *Pseudolabrys*, *Rhizomicrobium*, and *Rhodanobacter* from the phylum Proteobacteria, and *Jatrophihabitans* in the phylum Actinobacteria, when compared with the bare soil control (Figure 7). By contrast, three or more planted treatments exhibited significantly decreased proportions of *Achromobacter*, *Altererythrobacter*, *Arenimonas*, *Haliangium*, *Lysobacter*, *Massilia*, *Microvirga*, *Rubrivivax*, and *Sphingomonas* in the phylum Proteobacteria; *Aeromicrobium*, *Agromyces*, *Dactylosporangium*, *Geodermatophilus*, and *Lechevalieria* in the phylum Actinobacteria; *Aridibacter* in the Acidobacteria phylum; *Niabella* and *Niastella* in the phylum Bacteroidetes; and *Gemmatirosa* in the phylum Gemmatimonadetes (Figure 7).

### 3.3. Functional Prediction by PICRUSt

To determine the functions of the observed soil bacteria, we performed community prediction analysis using PICRUSt. The nearest sequenced taxon index (NSTI) values were in the range of 0.167–0.186 for bare soil samples, 0.175–0.201 for the *C. dactylon* soil samples, 0.170–0.174 for the *C. zizanioides* soil samples, 0.172–0.181 for the *M. glyptostroboides* soil samples, and 0.169–0.214 for the *S. matsudana* soil samples. These results were close to the NSTI values of soil samples studied by Langille et al. [20] (average NSTI = 0.17), indicating high accuracies in the predictions.

By comparing against the Kyoto encyclopedia of genes and genomes (KEGG) database, we obtained six categories of biological metabolic pathways (primary functional level): metabolism, genetic information processing, environmental information processing, cellular processes, organismal systems, and human diseases. Metabolism, genetic information processing, and environmental information processing appeared to be the primary components, accounting for 51.10%–51.84%, 15.84%–16.41%, and 11.65%–12.98% of all components, respectively. A comparison of the functional predictions of soil bacterial communities from different samples showed that the predicted gene copy number of three primary functional levels of biological metabolic pathways, including metabolism, genetic information processing, and human disease in the assayed soil samples decreased in the following order: *M. glyptostroboides* > *S. matsudana* > *C. dactylon* > bare soil > *C. zizanioides.* The predicted gene copy number of the remaining three primary functional levels of biological metabolic pathways ranked the highest in *M. glyptostroboides* samples.

**Figure 8:**
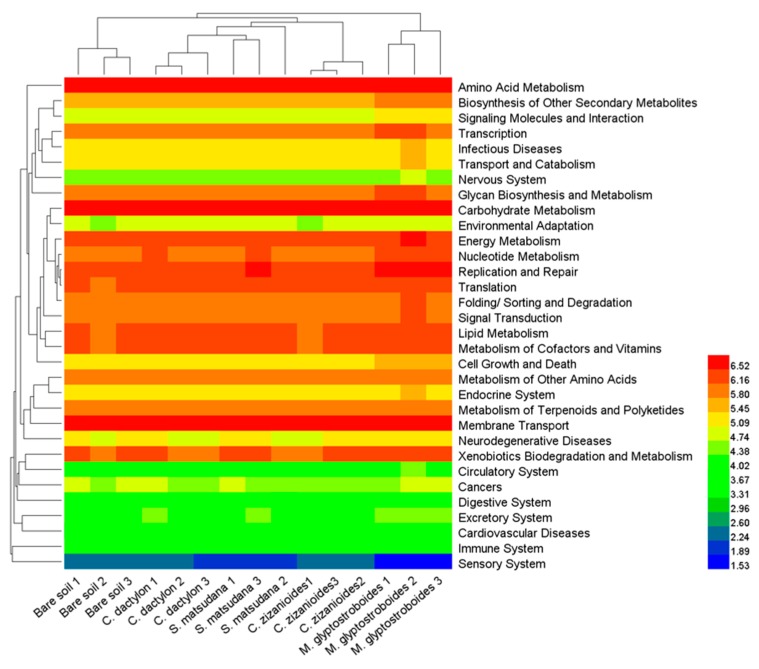
A heatmap showing the hierarchical clustering of the predicted Kyoto encyclopedia of genes and genomes (KEGG) Orthologs (KEGG level 2) gene copy number (log10 transformed) of bacterial microbiota across all samples. The digital number represents three biological replicates for each sample.

An analysis on the secondary functional levels of predicted genes identified 34 subfunctions, including the biosynthesis of other secondary metabolites, transcription, glycan biosynthesis and metabolism, and cell growth and death. Cluster analysis of the gene copy number (Figure 8) showed that the biological replications of each sample type clustered together, indicating good repeatability. The distances between the *S. matsudana*, *C. dactylon*, and *C. zizanioides* soil samples were close, while the bare soil and *M. glyptostroboides* soil samples were located far from the other samples. This was consistent with the UPGMA cluster analysis results on bacterial community compositions (Figure 4). The analysis on predicted gene copy number of the secondary functional levels showed that the predicted gene copy number for 11 subfunctions, such as carbohydrate metabolism, biosynthesis of other secondary metabolites, energy metabolism, and nucleotide metabolism decreased in the assayed soil samples in the following order: *M. glyptostroboides* > *S. matsudana* > *C. dactylon* > bare soil > *C. zizanioides*. The predicted gene copy number of seven subfunctions, such as glycan biosynthesis and metabolism, signal transduction, and cell growth and death decreased in the assayed soil samples in the following order: *M. glyptostroboides* > *S. matsudana* > *C. dactylon* > *C. zizanioides* > bare soil. The predicted gene copy number of six subfunctions such as metabolism of other amino acids, lipid metabolism, and digestive system decreased in the assayed soil samples in the following order: *M. glyptostroboides* > *S. matsudana* > bare soil > *C. dactylon* > *C. zizanioides*. 

**Figure 9:**
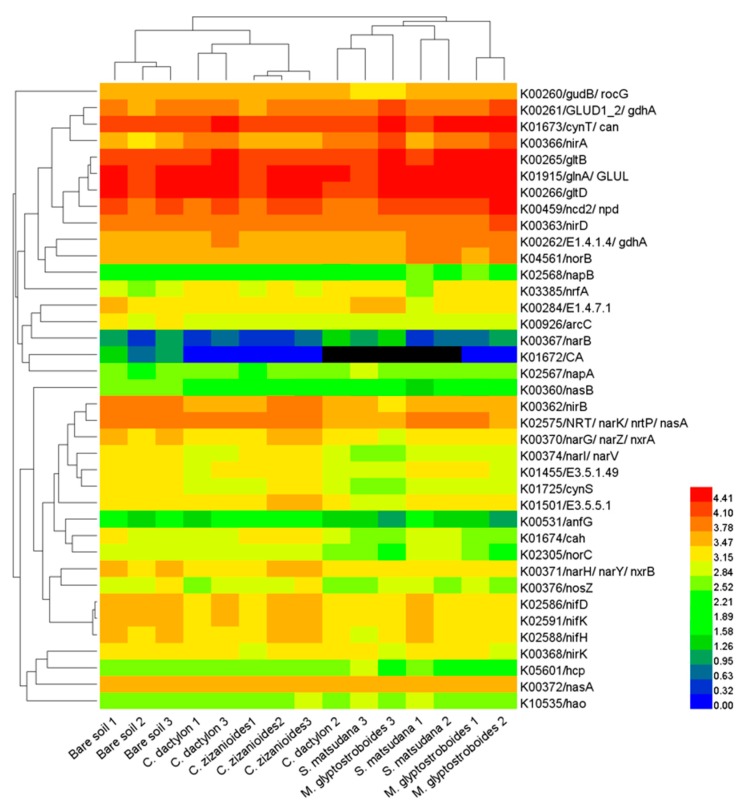
A heatmap showing the hierarchical clustering of the N metabolism related KOs based on predicted gene copy (log10 transformed) of bacterial microbiota across all samples. The digital number represents three biological replicates for each sample.

**Figure 10:**
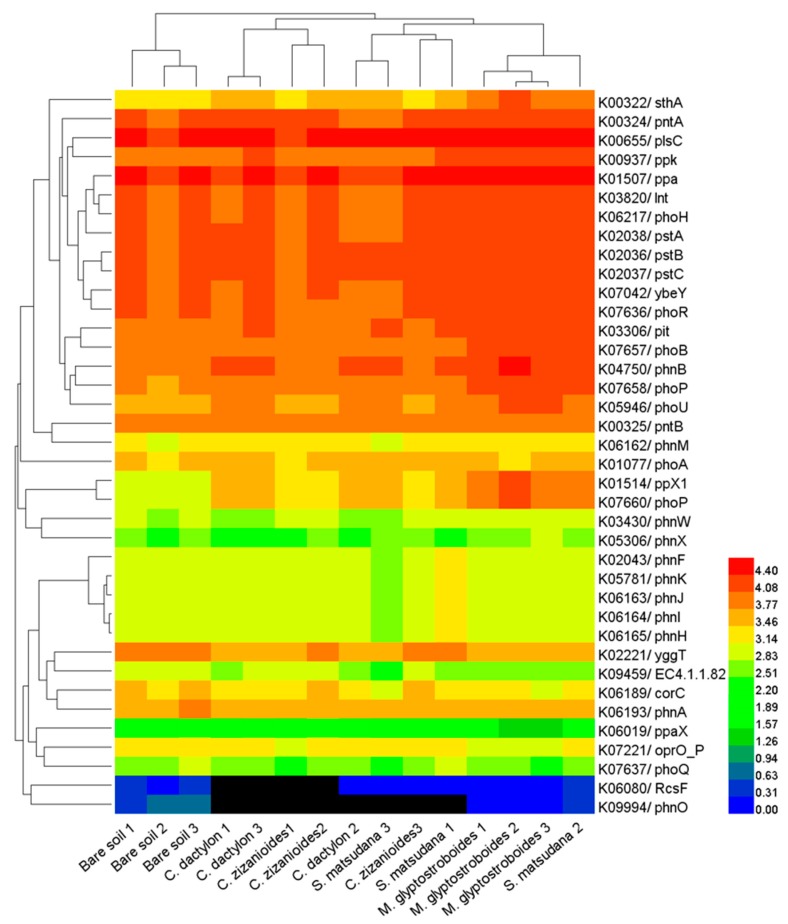
A heatmap showing the hierarchical clustering of the P metabolism related KOs based on predicted gene copy (log10 transformed) of bacterial microbiota across all samples. The digital number represents three biological replicates for each sample.

The Danjiangkou Reservoir has received large loads of N and P, and several studies have shown that PICRUSt can accurately predict the presence and abundance of functional genes in microbial communities [21,22,23]. Therefore, we analyzed N- and P-cycling-related functional genes in different samples from the WLFZ of the Danjiangkou Reservoir. By using PICRUSt, we predicted the relative abundances of some key N-cycling genes, primarily those involving N fixation (K02588, *nifH*), nitrification (K10535 *hao*), denitrification (K00370 *narG*, K00368 *nirK*, K04561 *norB*, and K00376 *nosZ*), and assimilatory and dissimilatory N reduction (K02575 *nasA*, K00367 *narB*, K02567 *napA*, K00366 *nirA*, K00362 *nirB*, and K03385 *nrfA*) (Figure 9). The cluster analysis results of the N-cycling gene copy number (Figure 9) showed that the *C. dactylon* and *C. zizanioides* soil samples clustered into one group, while those of the *S. matsudana* and *M. glyptostroboides* samples clustered into another group, with both clusters being separated from the bare soil samples. The above results indicate that the overall N metabolic capacity was similar between the *C. dactylon* and *C. zizanioides* soil samples, with a similar trend observed for the *S. matsudana* and *M. glyptostroboides* samples. The overall N metabolic capacity of the bare soil samples was different compared with those of the planted samples. P-cycling-related functional genes predicted by PICRUSt were compared to the results of LeBrun et al. [22]. PICRUSt predicted the relative abundance of some key P-cycling genes, primarily K00655 *plsC*, K01507 *ppa*, K02036 *pstB*, K02037 *pstC*, K02038 *pstA*, K00324 *pntA*, K06217 *phoH*, K03820 *lnt*, K07042 *ybeY*, and K07636 *phoR* (Figure 10). The cluster analysis results of P-cycling gene copy number were similar to those of the N metabolism-related analysis. The overall P metabolic capacity was similar between *C. dactylon* and *C. zizanioides* samples, similar to the trend observed for the *S. matsudana* and *M. glyptostroboides* samples. The overall P metabolic capacity of the bare soil samples was different from that of the other assayed samples.

## 4. Discussion

### 4.1. Bacterial Community Composition and Its Influence in the WLFZ of the Danjiangkou Reservoir

The water quality status in Danjiangkou Reservoir is directly related to drinking water safety for residents in the associated water-receiving area. Plants in the WLFZ can absorb and retain nutrients such as N and P and are important for protecting the water quality in the Danjiangkou Reservoir [9,10,11]. Microbes play an important role in this process, as they are primary drivers in the biogeochemical cycling of soil elements [12]. Although several studies investigated this process, due to the limitations of the traditional plate cultivation and clone library methods used, existing studies did not elucidate the microbial community composition and function with respect to plant absorption and retention of N and P in the WLFZ of the Danjiangkou Reservoir [10,13]. In the current study, we investigated soil from four artificially constructed plant communities, including herbs (*C. dactylon* and *C. zizanioides*), trees (*M. glyptostroboides*), and shrubs (*S. matsudana*), in the newly formed WLFZ of the Danjiangkou Reservoir. The community composition was analyzed by 16S rRNA gene sequencing using an Illumina MiSeq platform. Our results showed that the rhizosphere bacteria of plants in the Danjiangkou Reservoir WLFZ primarily comprised 24 phyla (e.g., Proteobacteria, Acidobacteria, Actinobacteria, and Gemmatimonadetes) and 362 genera (e.g., *Sphingomonas*, *Haliangium*, *Lysobacter*, *Gemmatimonas*, and *Massilia*), demonstrating the richness of the bacterial community composition at this site. In WLFZs, plants can absorb nutrients such as N and P, which are subsequently returned to the soil in the form of organic residues and aboveground litter, leading to changes in soil properties [6,9]. Measurements of soil physical and chemical properties showed that the rhizosphere soils of all four plants had higher organic matter and lower TN contents than those observed in the bare soil samples (Table 1). Plant roots provide sources of available C and N for rhizosphere soil microbes through root exudates and shedding, which results in significantly different biological properties in rhizosphere soils compared with those of nonrhizosphere or bare soils, which is referred to as the rhizosphere effect [35,36]. We analyzed the bacterial community composition in planted and bare soil samples by PCoA and UPGMA cluster analysis. Planting altered the bacterial community composition compared to bare soil, with varying effects observed among soil from the different plant types. Similar bacterial community compositions were observed in the *C. dactylon* and *S. matsudana* soil samples (Figure 3 and Figure 4). Analysis of different bacteria at the phylum and genus levels revealed that the different bacteria were primarily members of the Proteobacteria, Actinobacteria, Acidobacteria, and Bacteroidetes phyla. Significant changes occurred in the proportions of 25 genera (e.g., *Burkholderia*, *Paraburkholderia*, *Dokdonella*, *Achromobacter*, *Aeromicrobium*, and *Niabella*) for three or more planted soils compared with the bare soil control. These results indicate that the soil bacterial communities and soil properties were affected by artificially constructed vegetation in the newly formed WLFZ of the Danjiangkou Reservoir, with varying effects observed among soils associated with the different plant types.

### 4.2. Soil Bacterial Function and Influencing Factors in the WLFZ of the Danjiangkou Reservoir

The functional diversity of soil microbial communities is an important indicator for the soil microbial community and ecological function, and is of great significance for elucidating the role of microbial communities in different environments [37]. Metagenomics is an important approach used for the functional study of microbial communities, but its application is primarily constrained by its high cost [38]. The software PICRUSt, which was developed by Langille et al. [20] and is based on the use of 16S rRNA gene sequences, can predict the metabolic functional profile of the corresponding bacteria. The use of PICRUSt is advantageous in that it is a convenient, rapid procedure with a low cost. Furthermore, the metabolic functional profile prediction results of PICRUSt have high reliability. To date, this method has been used in numerous soil microbial studies [39,40]. Pii et al. [24] predicted the function of rhizosphere bacterial communities in barley and tomato using PICRUSt, observing that metabolism was a major component of the functional modules, while some OTUs were closely related to functions such as C fixation and N and S metabolism. Currently, functional studies of soil microbial communities in WLFZs are rarely reported. To elucidate the function of microbial communities in the nutrient adsorption and retention by plants in the WLFZ of the Danjiangkou Reservoir, we performed a PICRUSt functional prediction analysis on the high-throughput sequencing data generated in this study. Similar to the results of Pii et al. [24], we observed that soil bacteria in the WLFZ of the Danjiangkou Reservoir were primarily involved in six biological metabolic pathways (e.g., metabolism, genetic information processing, and environmental information processing), among which metabolism was a major component. Compared with the bare soil samples, the planted soil samples of *M. glyptostroboides*, *S. matsudana*, and *C. dactylon* had higher predicted gene copy numbers at the primary and secondary functional levels, indicating a higher metabolic capacity of bacteria in these planted samples. Our results agree with those from a study by Mendes et al. [41], who investigated the function of soybean rhizosphere bacteria and showed that soybean planting elevated the expression levels of functional gene groups involved in "membrane transport", "N metabolism", and "P metabolism". LeBrun et al. [22] showed that PICRUSt can accurately predict the presence and abundance of functional genes, and a comparison revealed its higher accuracy relative to microarray analysis. Thus, the use of PICRUSt appears appropriate when asking questions specifically related to microbial community N and P metabolism, as was performed in this study. Our results show that PICRUSt predicted the relative abundances of some key N-cycling genes, primarily those involved in N fixation, nitrification, and denitrification (Figure 9). We also predicted the relative abundances of some key P-cycling genes, including *plsC*, *ppa*, *pstB*, *pstC*, *pstA*, and *pntA* (Figure 10). Cluster analysis of these N- and P-cycling gene copy numbers revealed that planting altered the N- and P-cycling metabolic capacity of the soil bacterial community, with variation observed among bacterial communities associated with the different plant species. The overall N- and P-cycling metabolic capacity was similar for the *C. dactylon* and *C. zizanioides* samples, as well as for the *S. matsudana* and *M. glyptostroboides* soil samples. Based on the PICRUSt analysis of bacterial community functions, we conclude that planting improved the function of bacteria and especially altered their N- and P-cycling metabolic capacity in the WLFZ of the Danjiangkou Reservoir.

## 5. Conclusions

An analysis of rhizosphere and bare soil samples from the WLFZ of the Danjiangkou Reservoir showed that the soil physiochemical properties and bacterial community compositions were altered by the artificially planting of *C. zizanioides*, *C. dactylon*, *M. glyptostroboides*, and *S. matsudana*. A PICRUSt analysis revealed that planting also improved the metabolic capacity of bacteria and changed their N- and P-cycling metabolic capacity, which varied by plant type. This study presents a preliminary analysis on differences in N and P depletion associated with various plants from the perspective of associated microbial communities and provides a reference for vegetation construction in the WLFZ of the Danjiangkou Reservoir. However, the seasonal dry–wet alternation in the WLFZ can cause intense environmental changes. Because the present study was conducted during the dry period, changes associated with other periods of the year need to be further investigated.

## Figures and Tables

**Table 1:** Main physicochemical characteristics of soil samples (means of three replicates ± standard errors).

Samples	pH	TN (g/kg)	TP (g/kg)	TK (g/kg)	NO_3_^−^–N (mg/kg)	NH_4_^+^–N (mg/kg)	SOC (g/kg)
Bare soil	8.61 ± 0.20d	1.34 ± 0.07d	0.79 ± 0.03c	4.95 ± 0.22bc	123.80 ± 4.00c	79.35 ± 1.96b	25.60 ± 2.24a
*C. dactylon*	8.16 ± 0.24c	0.90 ± 0.04b	0.82 ± 0.02c	4.51 ± 0.24a	133.26 ± 4.40c	77.93 ± 1.99b	32.77 ± 2.63b
*C. zizanioides*	8.37 ± 0.17cd	0.36 ± 0.02a	0.71 ± 0.02b	4.47 ± 0.24a	56.39 ± 3.19a	71.60 ± 2.02a	30.29 ± 2.88b
*M. glyptostroboides*	7.15 ± 0.14a	1.09 ± 0.09c	0.66 ± 0.03ab	5.15 ± 0.16c	94.00 ± 3.87b	92.61 ± 3.48c	30.92 ± 2.07b
*S. matsudana*	7.74 ± 0.17b	0.44 ± 0.01a	0.65 ± 0.03a	4.75 ± 0.13ab	128.70 ± 8.23c	75.55 ± 4.19ab	30.77 ± 1.16b

Means within the same column followed by the same letter are not significantly different at *p* < 0.05, as based on one-way ANOVA.

**Table 2:** Estimation of bacterial community diversity (means of three replicates ± standard errors).

Samples	Reads	sobs	Shannon indices	Simpson indices	ACE indices	Chao 1 indices	Coverage (%)
Bare soil	34,367 ± 4943	2741 ± 264b	6.48 ± 0.13bc	0.0058 ± 0.0011ab	3489 ± 258a	3494 ± 259b	97.64 ± 0.31
*C. dactylon*	30,682 ± 3827	2451 ± 98ab	6.46 ± 0.06bc	0.0047 ± 0.0005a	3235 ± 108ab	3246 ± 133ab	97.48 ± 0.27
*C. zizanioides*	32,300 ± 3305	2672 ± 107b	6.52 ± 0.04c	0.0047 ± 0.0003a	3525 ± 138a	3539 ± 236b	97.37 ± 0.22
*M. glyptostroboides*	36,564 ± 3704	2414 ± 137ab	6.10 ± 0.14a	0.0065 ± 0.0015b	3274 ± 120ab	3281 ± 149ab	97.79 ± 0.19
*S. matsudana*	32,369 ± 3233	2283 ± 199a	6.30 ± 0.14ab	0.0054 ± 0.0005ab	3005 ± 242a	3005 ± 294a	97.80 ± 0.35

Means within the same column followed by the same letter are not significantly different at *p* < 0.05, as based on one-way ANOVA. ACE–abundance-based coverage estimator, sobs–total number of species observed.
